# The age-specific comorbidity burden of mild cognitive impairment: a US claims database study

**DOI:** 10.1186/s13195-023-01358-8

**Published:** 2023-12-06

**Authors:** Gang Li, Nicola Toschi, Viswanath Devanarayan, Richard Batrla, Tommaso Boccato, Min Cho, Matteo Ferrante, Feride Frech, James E. Galvin, David Henley, Soeren Mattke, Susan De Santi, Harald Hampel

**Affiliations:** 1 Eisai Inc., 200 Metro Boulevard, Nutley, NJ 07110 USA; 2 Department of Biomedicine and Prevention, University of Rome Tor Vergata, Via Montpellier 1, 00133 Rome, Italy; 3 A.A. Martino’s Center for Biomedical Imagin, Harvard Medical School, Boston, USA; 4 Miller School of Medicine, University of Miami, 7700 W Camino Real, Suite 200, Boca Raton, FL 33433 USA; 5 Research and Development, Janssen Pharmaceuticals, Inc., 1125 Bear Tavern Rd, Titusville, NJ 08560 USA; 6 The USC Brain Health Observatory, University of Southern California, 635 Downey Way, Los Angeles, CA 90089 USA

**Keywords:** Mild cognitive impairment (MCI), MCI risk prediction, MCI comorbidities, Age group

## Abstract

**Background:**

Identifying individuals with mild cognitive impairment (MCI) who are likely to progress to Alzheimer’s disease and related dementia disorders (ADRD) would facilitate the development of individualized prevention plans. We investigated the association between MCI and comorbidities of ADRD. We examined the predictive potential of these comorbidities for MCI risk determination using a machine learning algorithm.

**Methods:**

Using a retrospective matched case-control design, 5185 MCI and 15,555 non-MCI individuals aged ≥50 years were identified from MarketScan databases. Predictive models included ADRD comorbidities, age, and sex.

**Results:**

Associations between 25 ADRD comorbidities and MCI were significant but weakened with increasing age groups. The odds ratios (MCI vs non-MCI) in 50–64, 65–79, and ≥ 80 years, respectively, for depression (4.4, 3.1, 2.9) and stroke/transient ischemic attack (6.4, 3.0, 2.1). The predictive potential decreased with older age groups, with ROC-AUCs 0.75, 0.70, and 0.66 respectively. Certain comorbidities were age-specific predictors.

**Conclusions:**

The comorbidity burden of MCI relative to non-MCI is age-dependent. A model based on comorbidities alone predicted an MCI diagnosis with reasonable accuracy.

**Supplementary Information:**

The online version contains supplementary material available at 10.1186/s13195-023-01358-8.

## Background

Mild cognitive impairment (MCI) is a condition associated with memory loss and cognitive deficits beyond what is expected with normal aging and may be a transitional stage before Alzheimer’s disease and related dementias (ADRD) [1]. For individuals with MCI, the likelihood of progression to any form of dementia is estimated to occur at a rate three to five times higher than among those without MCI [2–5]. The prevalence of MCI in the United States (US) increases with age, ranging from about 7% of people aged 60 to 64 to about 25% of people aged 80 to 84 [6].

While MCI could be identified as part of regular care by primary care physicians (PCPs), studies have found that the detection of MCI is limited and published identification rates of MCI by PCPs are as low as six to 15% [7, 8]. PCPs’ perceived barriers to detection include patients who do not disclose symptoms and family members assuming that symptoms are a natural part of aging [9]. Additional reasons for missed opportunities to identify MCI individuals include the lack of widely used cognitive assessment tools, lack of training on cognitive assessments, inconsistent opportunities for screening, lack of easily accessible biomarkers and imaging tests, and limited time during patient visits [10, 11].

Timely identification of MCI is important to facilitate an individualized management plan that addresses underlying conditions and possibly slows the progression to ADRD [12, 13]. The Food & Drug Administration (FDA) has recently approved two drugs that target the underlying pathology of AD by aiming to remove amyloid-beta plaques from the brain. These medicines are indicated for treatment in the early stages of disease [14, 15]. Cummings et al. analyzed information on clinicaltrials.gov and found that among the 143 agents in development for AD treatment, more than half of the phase 3 studies included participants with preclinical AD, MCI, or mild AD [16].

The barriers to early identification point to the utility and value of a tool that could help PCPs detect warning signs of MCI, in particular in younger age groups with a lower prevalence. Such a tool could use electronic health records (EHR) data to estimate the risk of developing MCI based on the presence of comorbidities. Such a tool would facilitate the detection of MCI, leading to potential treatment. Other studies have focused on identifying medical risk factors for developing AD [17] or ADRD [18–24]. Machine learning models using a large dataset of medical claims and EHR data have been shown to be effective in predicting the onset of dementia [25]. Our study expands previous research by using traditional statistical and machine learning models to explore the risk for MCI in individuals with established ADRD comorbidities by age group.

## Methods

### Data source and study design

This non-interventional retrospective matched case-control study used the Merative MarketScan Commercial and Medicare Databases and had an observation period from January 01, 2014, through December 31, 2019. These databases represent the health services of employees, dependents, and retirees in the US with primary or Medicare coverage through privately insured fee-for-service, point-of-service, or capitated health plans. The Commercial and Medicare Databases are generally representative of the population in the US in terms of gender. All enrollment records and inpatient, outpatient, ancillary, and drug claims were collected and used for study population identification as well as outcomes measurements.

As the study did not involve interactions with or interventions among human subjects and all data were de-identified per US federal regulations (45 CFR 46, 102(f))20, it was exempt from institutional review board (IRB) review, consent requirements and registration.

### Study population

This study compared a cohort of individuals with an MCI diagnosis, but without an ADRD diagnosis at entry, to a cohort without an MCI or ADRD diagnosis. The index date for each individual in the MCI cohort was the date of the first MCI diagnosis. Individuals in the non-MCI cohort were matched three to one to an individual in the MCI cohort using age, sex, geographic region, and the year in which the individual had data entered into the MarketScan databases via the propensity score method. The index dates for non-MCI individuals were the index dates of their matched MCI individuals.

The pre-index period was defined as 2 years before the index date, and the follow-up period was defined as the time from the index date to the last visit recorded (minimum of one year of follow-up required). All individuals in the study were required to be at least 50 years old in the year of the index date. An MCI diagnosis was defined by an International Classification of Disease (ICD) code, either ICD-9 code 331.83 or ICD-10 code G31.84. To be included in the non-MCI cohort, individuals could not have an MCI or ADRD diagnosis throughout their inclusion in the MarketScan databases. All individuals were excluded if they (1) received a diagnosis of Parkinson’s disease at any time or (2) received donepezil, memantine, memantine/donepezil, galantamine, or rivastigmine during the pre-index period [26].

### Study measures

Individuals in the MCI and non-MCI cohorts were categorized by age group (50 to 64 years, 65 to 79 years, 80+ years). Most individuals are eligible for Medicare at age 65, so 15-year increments were chosen to define age groups. Baseline demographic and clinical characteristics assessed included age at index, sex, region, Charlson Comorbidity Index (CCI), Elixhauser Comorbidity Index (ECI), ADRD comorbidities, and baseline treatments for non-AD-related conditions [27].

A literature review was conducted to identify ADRD comorbidities [28–30]. Twenty-seven ADRD comorbidities were identified, which will be simply referred to as comorbidities. These were categorized and included cardiovascular diseases (hypertension, stroke/transient ischemic attack [TIA], ischemic heart disease, congestive heart failure, myocardial infarction, atherosclerosis, atrial fibrillation), metabolic disorders (diabetes, hyperlipidemia, obesity, metabolic syndrome, weight loss), psychiatric disorders (depression, insomnia, bipolar, schizophrenia, psychosis, alcohol abuse, drug abuse), and other diseases (chronic kidney disease, chronic periodontitis, chronic pulmonary disease, hearing loss, obstructive sleep apnea, disturbances of sensation of smell and taste, hypothyroidism, traumatic brain injuries/concussion). Indices of these comorbidities and their treatments within 2 years before the index date were derived using ICD-9 and ICD-10 codes from both inpatient and outpatient visits [27]. Chronic periodontitis and traumatic brain injuries/concussion were excluded from the analysis because less than 0.3% of individuals with MCI had either condition.

### Statistical analysis

Continuous variables were reported as mean (standard deviation [SD]) and median (lower quartile [Q1]–upper quartile [Q3]) and range by cohort, and subgroups in the MCI and non-MCI cohorts were compared using an independent *t*-test. Categorical variables were reported as frequencies and percentages, and cohorts, and subgroups in the MCI and non-MCI cohorts were compared using a chi-square test.

Odds ratios (ORs, MCI vs non-MCI) and two-sided 95% confidence intervals (CIs) were calculated based on percentages for each comorbidity for all patients and each age group. If the lower bound of the 95% CI was greater than one, it would indicate a statistically significant association between MCI and comorbidity. Furthermore, the ORs of each comorbidity among age groups were compared using a logistic regression model that included the age by comorbidity interaction term, including one comorbidity at a time. If a *p*-value for the age by comorbidity interaction term was less than 0.05, the ORs between the age groups would be considered statistically significantly different.

To predict the risk for MCI, several machine learning approaches (Bayesian logistic lasso regression [BLLR] with a mixture of double-exponential prior [31], stochastic gradient boosting machine, extreme gradient boosting [XGBoost], and regularized random forests) were applied. The area under the curve (AUC) of the receiver operating characteristic (ROC) curve, which was referred to as ROC-AUC, was used to evaluate each model. For machine-learning approaches, the model included age, sex, and 25 comorbidities as predictors; a subset of ADRD comorbidities was automatically selected by these algorithms, with the appropriate weight given based on their relative influence on the prediction. Training and test sets were created via a random 70–30% split, stratified by MCI/non-MCI status and by age. The MCI/non-MCI imbalance was accounted for in the prediction modeling algorithm by using the prevalence rate as the probability cutoff. Prediction performance was first evaluated within the 70% training set via ten iterations of ten-fold stratified cross-validation followed by evaluation in the 30% test set via the AUC of the ROC (ROC-AUC) [32]. Results from the best-performing algorithm are reported. The ROC-AUCs were compared via a bootstrap testing procedure [33].

All descriptive analyses were performed using SAS statistical software (version 9.4). The machine learning approaches were performed using R (version 4.2.2) [34].

## Results

### Demographics and clinical characteristics of the two cohorts at baseline

A total of 5185 individuals with an MCI diagnosis and 15,555 matched non-MCI individuals met the eligibility criteria. The mean age at index was 67 years for both the MCI and non-MCI cohorts, and 57.7% of individuals were female (Table 1). Mean CCI and ECI were higher in the MCI cohort compared to the non-MCI cohort (1.5 vs. 1.0 and 2.6 vs. 1.8, respectively; *p* < .001) (Table 1).

**Table 1:** Patient demographics, clinical characteristics, and ADRD comorbidities in the pre-index period—overall population

	**MCI**		**Non-MCI**		
	***N***	**%**	***N***	**%**	
**Baseline characteristics**					*P-*value*
**Total**	5185	100.0%	15,555	100.0%	
**Age at index (continuous)**					
Mean (SD)	67.0 (12.0)		67.0 (12.0)		.950
Median (Q1–Q3)	63.0 (57.0 - 77.0)		63.0 (57.0 - 77.0)		
Min–max	50 - 101		50 - 116		
**Age (categorical)**					
50–64	2781	53.6%	8343	53.6%	1.00
65–79	1375	26.5%	4125	26.5%	
≥ 80	1029	19.8%	3087	19.8%	
**Sex**					
Male	2194	42.3%	6582	42.3%	1.00
Female	2991	57.7%	8973	57.7%	
**Region**					
Northeast	1571	30.3%	4664	30.0%	.145
North Central	877	16.9%	2647	17.0%	
South	1924	37.1%	5825	37.4%	
West	806	15.5%	2407	15.5%	
Other/unknown	7	0.1%	12	0.1%	
**Charlson Comorbidity Index**					
Mean (SD)	1.5 (1.57)		1.0 (1.29)		<.001
Median (Q1–Q3)	1.0 (0.0 - 2.0)		0.0 (0.0 - 1.0)		
Min–Max	0 - 9		0 - 10		
**Elixhauser Index**					
Mean (SD)	2.6 (2.1)		1.8 (1.87)		<.001
Median (Q1–Q3)	2.0 (1.0 - 4.0)		1.0 (0.0 - 3.0)		
Min–Max	0 -13		0 - 13		
**ADRD comorbidities**					Odds Ratio (95% CI)**
Patients with ≥1 any condition	4956	95.6%	12,668	81.4%	4.9 (4.3, 5.7)
Hypertension	3379	65.2%	8606	55.3%	1.5 (1.4, 1.6)
Stroke/TIA	1091	21.0%	1167	7.5%	3.3 (3.0, 3.6)
Ischemic heart disease	1137	21.9%	2204	14.2%	1.7 (1.6, 1.8)
Congestive heart failure	415	8.0%	839	5.4%	1.5 (1.4, 1.7)
Myocardial infarction	231	4.5%	438	2.8%	1.6 (1.4, 1.9)
Atherosclerosis	519	10.0%	1078	6.9%	1.5 (1.3, 1.7)
Atrial fibrillation	498	9.6%	1082	7.0%	1.4 (1.3, 1.6)
Diabetes	1309	25.2%	3012	19.4%	1.4 (1.3, 1.5)
Hyperlipidemia	3423	66.0%	8610	55.4%	1.6 (1.5, 1.7)
Obesity	928	17.9%	2116	13.6%	1.4 (1.3, 1.5)
Metabolic syndrome	276	5.3%	401	2.6%	2.1 (1.8, 2.5)
Weight loss	320	6.2%	362	2.3%	2.8 (2.4, 3.2)
Hearing loss	1090	21.0%	1746	11.2%	2.1 (1.9, 2.3)
Depression	1622	31.3%	1683	10.8%	3.8 (3.5, 4.1)
Insomnia	758	14.6%	941	6.0%	2.7 (2.4, 2.9)
Obstructive sleep apnea	1440	27.8%	1825	11.7%	2.9 (2.7, 3.1)
Disturbances of sensation of smell and taste	35	0.7%	24	0.2%	4.4 (2.6, 7.4)
Bipolar	120	2.3%	55	0.4%	6.7 (4.8, 9.2)
Schizophrenia	21	0.4%	13	0.1%	4.9 (2.4, 9.7)
Psychosis	73	1.4%	27	0.2%	8.2 (5.3, 12.8)
Alcohol abuse	136	2.6%	158	1.0%	2.6 (2.1, 3.3)
Drug abuse	142	2.7%	141	0.9%	3.1 (2.4, 3.9)
Hypothyroidism	1298	25.0%	2717	17.5%	1.6 (1.5, 1.7)
Chronic kidney disease	204	3.9%	379	2.4%	1.6 (1.4, 2.0)
Chronic pulmonary disease	1257	24.2%	2561	16.5%	1.6 (1.5, 1.8)

*Abbreviations: ADRD* Alzheimer’s disease and related dementia disorders, *CI* confidence interval, *Max* maximum, *MCI* mild cognitive impairment, *Min* minimum, *OR* odds ratio, *Q1* 1st quartile, *Q3* 3rd quartile, *SD* standard deviation, *TIA* transient ischemic attack

^*^p-values resulted from independent *t*-tests conducted for continuous variables and chi-square tests for categorical variables

^**^ORs and 95% confidence intervals were calculated directly from percentages and frequencies for MCI and non-MCI cohorts

Twenty-five ADRD comorbidities were found to occur in statistically significantly higher proportions in MCI individuals vs. non-MCI individuals, with the lower 95% CI limits of the ORs greater than one (Table 1). The MCI cohort had a statistically significantly higher frequency of patients with one or more of the 25 comorbidities (95.6% vs. 81.4%) with OR (MCI vs non-MCI, 95% CI) of 4.9 (4.3, 5.7) (Table 1).

The 15 comorbidities with the largest frequency in the MCI cohort were as follows: hyperlipidemia (66.0%), hypertension (65.2%), depression (31.3%), obstructive sleep apnea (27.8%), diabetes (25.2%), hypothyroidism (25.0%), chronic pulmonary disease (24.2%), ischemic heart disease (21.9%), stroke/TIA (21.0%), hearing loss (21.0%), obesity (17.9%), insomnia (14.6%), atherosclerosis (10.0%), atrial fibrillation (9.6%), and congestive heart failure (8.0%) (Table 1).

The 15 comorbidities with the largest OR (MCI vs. non-MCI) were as follows: psychosis with an OR (95% CI) of 8.2 (5.3, 12.8), bipolar 6.7 (4.8, 9.2), schizophrenia 4.9 (2.4, 9.7), disturbances of sensation of smell and taste 4.4 (2.6, 7.4), depression 3.8 (3.5, 4.1), stroke/TIA 3.3 (3.0, 3.6), drug abuse 3.1 (2.4, 3.9), obstructive sleep apnea 2.9 (2.7, 3.1), weight loss 2.8 (2.4, 3.2), insomnia 2.7 (2.4, 2.9), alcohol abuse 2.6 (2.1, 3.3), hearing loss 2.1 (1.9, 2.3), metabolic syndrome 2.1 (1.8, 2.5), ischemic heart disease 1.7 (1.6, 1.8), and hyperlipidemia 1.6 (1.5, 1.7) (Table 1).

Seven comorbidities appeared on both lists of the highest frequency and highest OR. These seven were presented in the order of OR as follows: depression, stroke/TIA, obstructive sleep apnea, insomnia, hearing loss, ischemic heart disease, and hyperlipidemia.

**Fig. 1:**
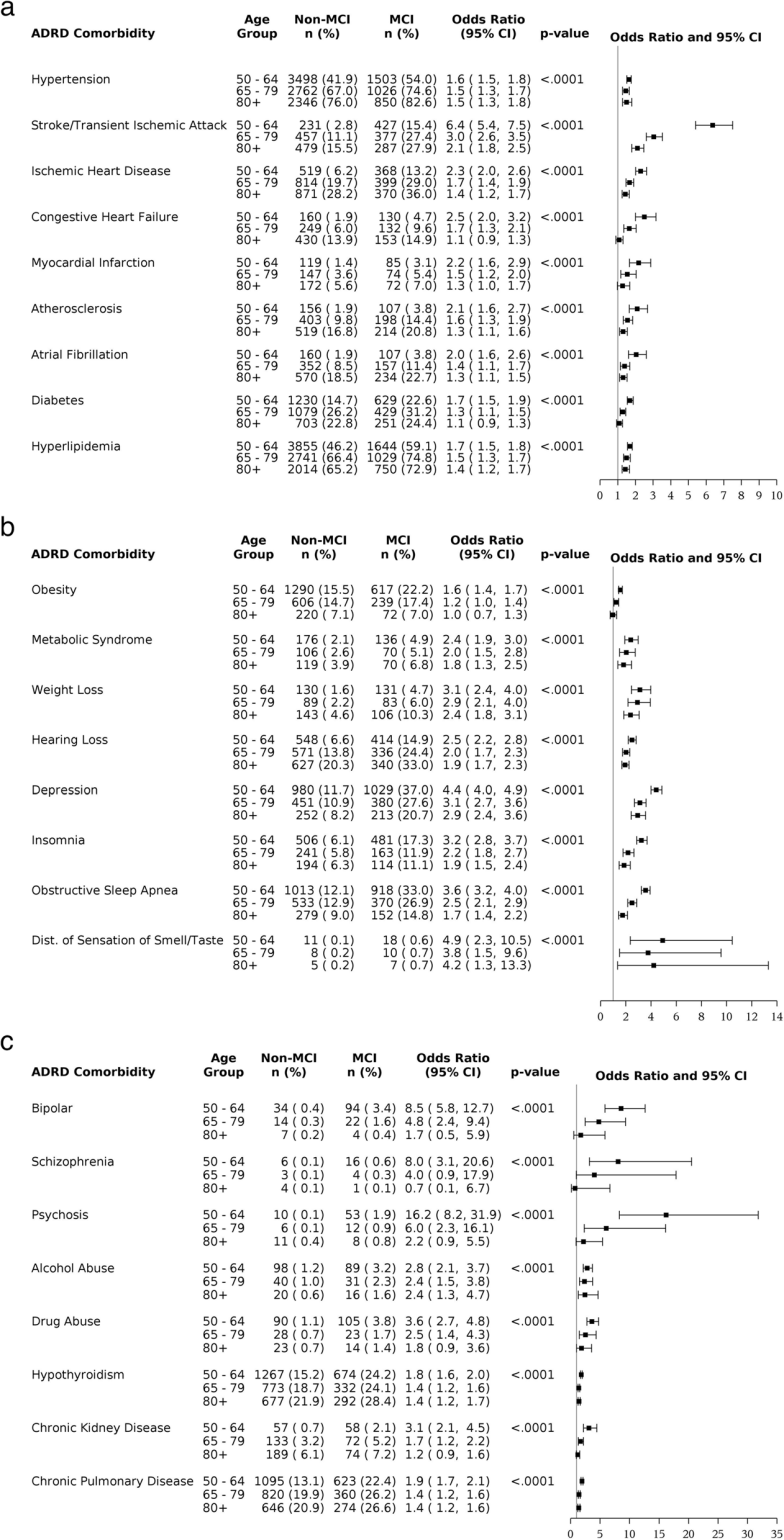
Forest plot of the prevalence odds ratio of ADRD comorbidities at baseline by age group. Note. ORs and 95% confidence intervals were calculated directly from percentages and frequencies for MCI and non-MCI cohorts in each age group. The *p*-values were for testing the equality of ORs among age groups and obtained by assessing comorbidity by age group interaction in the logistic regression model with cohort as response variable and the comorbidity, age group, and the interaction of the comorbidity by age group

When compared by age group, the OR (MCI vs non-MCI) decreased significantly with increasing age groups across the 25 comorbidities (Fig. 1). For depression, the OR was statistically significantly higher for the age 50 to 64 age group (4.4 [4.0, 4.9]) compared with the 65 to 79 age group (3.1 [2.7, 3.6]) and 80+ age group (2.9 [2.4, 3.6]) (*p* < .05). For stroke/TIA, the OR was significantly greater in the 50 to 64 years age group (6.4 [5.4, 7.5]) compared with 65 to 79 years (3.0 [2.6, 3.5]) and 80+ years (2.1 [1.8, 2.5]) with *p* < .05.

### MCI risk prediction models

As the other machine-learning algorithms such as stochastic gradient boosting and random forests yielded similar prediction performance with the ROC-AUCs ranging from 74 to 76% and were not significantly different from the BLLR algorithm (*p* > 0.05, based on bootstrap test) [33], the results from only the BLLR algorithm are reported in this paper due to its simplicity and ease of interpretation. The BLLR had ROC-AUC 0.72 (Table 2) for the analysis of all individuals; age was a significant (*p* <.001) predictor while sex was not.

**Table 2:** Performance of Bayesian logistic lasso regression for all individuals and by age group

	**Test set (30% hold out set)**		**ROC-AUC**
**Group**	**Sensitivity**	**Specificity**	
**All individuals**	59.9%	73.8%	0.72
**Age 50–64 years**	61.9%	77.4%	0.75
**Age 65–79 years**	59.3%	71.7%	0.70
**Age ≥ 80 years**	55.3%	66.8%	0.66

*ROC-AUC*, area under the curve of the receiver operating characteristic

Performance of the model for MCI risk prediction varied widely across the three age groups with ROC-AUC 0.75, 0.70, and 0.66 for the age groups 50 to 64 years, 65 to 79 years, and 80+ years, respectively (Table 2). The ROC-AUC value for the 50 to 64 years age group was statistically significantly higher than both the 65 to 79 age group (*p* < .05) and the 80+ age group (*p* < .05). However, the difference between the ROC-AUC values for the 65 to 79 age group and the 80+ age group was not statistically significantly different (*p* = .1299). The sensitivity and specificity are presented in Table 2. The 50 to 64 years group had the best performance with a sensitivity of 61.9% and a specificity of 77.4%; the 65–79 years group had a sensitivity of 59.3% and a specificity of 71.7%; the 80+ years had the least performance with a sensitivity of 55.3% and a specificity of 66.8%.

**Fig. 2:**
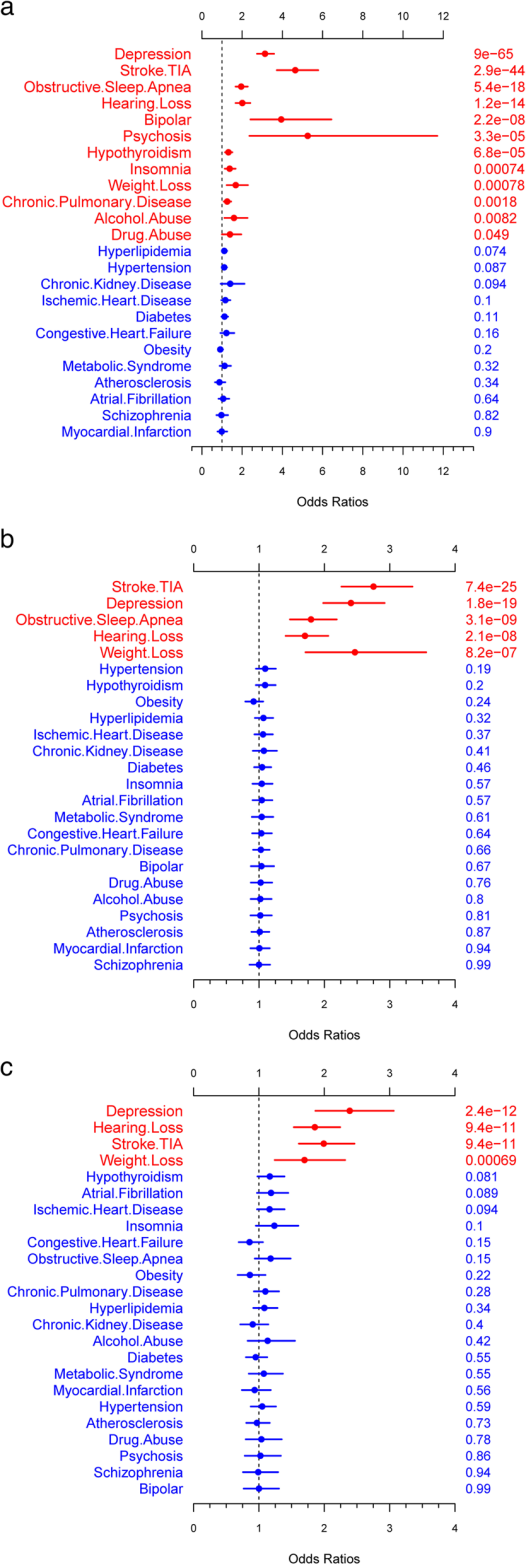
ADRD comorbidities of Bayesian logistic lasso regression model by age group. **a** Age group 50–64 years. **b** Age group 65–79 years. **c** Age group 80+ years

The BLLR identified the comorbidities that were significant predictors of MCI for each age group (Fig. 2). For age group 50 to 64 years, 12 comorbidities were significant predictors of MCI diagnosis in the multivariate model with *p*-values < 0.05: depression, stroke/TIA, obstructive sleep apnea, hearing loss, bipolar, psychosis, hypothyroidism, insomnia, weight loss, chronic pulmonary disease, alcohol abuse, and drug abuse. For the age group 65 to 79 years, five comorbidities significantly impacted the model with *p*-values < 0.05: stroke/TIA, depression, obstructive sleep apnea, hearing loss, and weight loss. For the age group 80+ years, four comorbidities significantly impacted the model with *p*-values < 0.05: depression, hearing loss, stroke/TIA, and weight loss.

## Discussion

The results of this retrospective study of the MarketScan Commercial and Medicare Databases suggest that ADRD comorbidities are also comorbidities for MCI. The BLLR algorithm was selected due to its simplicity and interpretability and because it yielded similar prediction performance as the other machine-learning algorithms.

The 25 ADRD comorbidities identified by our literature search were also significant risk factors for MCI in this population. Individuals in the MCI cohort had a higher frequency of comorbidities compared with the non-MCI cohort. The differences between cohorts for depression and stroke/TIA were the largest. Depression, stroke/TIA, obstructive sleep apnea, insomnia, hearing loss, ischemic heart disease, and hyperlipidemia appeared on both the list of the highest frequency comorbidities and the list of comorbidities with the highest ORs, suggesting these seven comorbidities may be predictive of MCI. Considering that MCI is a prodromal stage for ADRD, the connection between ADRD comorbidities and MCI comorbidities is not unexpected; however, identifying the comorbidities with the strongest connections and quantifying the relationships can help inform the development of a screening tool to identify high-risk individuals.

Secondly, the ORs (MCI vs. non-MCI) decreased with increasing age group for all comorbidities. The differences were statistically significantly higher for the age 50 to 64 group compared with both older age groups. As a result, the potential for ADRD comorbidities to predict MCI risk also declined with increasing age group. The BLLR results in this study demonstrated better model predictivity in the younger age group. Depression, stroke/TIA, hearing loss, and weight loss were significant predictors across all age groups; however, obstructive sleep apnea was significant only for the two youngest age groups. Furthermore, hypothyroidism, insomnia, bipolar, chronic pulmonary disease, psychosis, alcohol abuse, and drug abuse were only significant for the youngest age group.

Changes in the likelihood of observing a particular comorbidity in different age groups may be related to the epidemiology of the specific condition. For cardiovascular and metabolic diseases, frequencies increased by age group in both the MCI and non-MCI cohorts; thus, ORs decreased. This aligns with the literature because people are more likely to develop cardiovascular and metabolic diseases with increasing age [35–37]. The association between hypertension in midlife, which aligns with the youngest age group for this study, and cognitive decline has been well established [38, 39]. The relationship between hypertension developing in late life and dementia is less clear. A 24-year prospective study found that in addition to hypertension in midlife and late life, a history of hypertension followed by late-life hypotension was also associated with an increased risk of dementia [40]. Our study evaluates hypertension over a shorter time period but confirms the importance of considering the temporal effects of comorbidities.

The frequency of psychiatric disorders, including depression and bipolar, decreased in the oldest age group in both cohorts. This aligns with the literature because the prevalence of psychiatric disorders decreases with age, in part because of the reduction in life expectancy of people with depression [41].

The observed differences in the oldest and youngest age groups in this study may have been impacted by survivorship bias. To reach the oldest age group, individuals likely maintained good health during the prior years. The number of years with comorbidity is likely to impact the onset of dementia and longevity [42].

Another possible contributor to lower ORs in the older age groups in this study may be the effect of undiagnosed MCI in the non-MCI cohort. The proportion of undiagnosed MCI individuals is likely similar to MCI prevalence at the population level, which increases with age. For people aged 50 to 64 years, the MCI prevalence is about 6.7% [4]. If our cohorts are similar to the general population, the non-MCI cohort may include a similar percentage of undiagnosed MCI individuals, which should have little impact on the frequencies for comorbidities in the non-MCI cohort and the ORs. However, for the 80+ years group, the MCI prevalence in the general population is about 25.2% [4]; the impact of undiagnosed MCI on ORs in this age group may not be ignored.

Our findings suggest that chronic pulmonary disease is a predictor of MCI; however, reports of the association between chronic obstructive pulmonary disease and ADRD outcomes are conflicting and limited [35, 36]. Thus, additional research on the association between chronic pulmonary disease and MCI and ADRD is recommended, as well as further evaluation to quantify the impact of smoking on MCI.

Chronic periodontitis and head injury were excluded from this analysis because fewer than 0.3% of individuals received a diagnosis. For chronic periodontitis, this may be because dentists were most likely to diagnose and treat that condition. MarketScan data do not capture dental records. A diagnosis of chronic periodontitis would only be captured in the dataset if it were noted in a medical office setting. For head injury, a longer baseline period may be required to quantify the relationship.

According to the package inserts for aducanumab and lecanemab-irmb, treatment should be initiated in patients with MCI or at the mild dementia stage of disease [14, 15]. With the availability of new treatments and screening tools for mild and early-stage ADRD, clinical guidelines will need more frequent updates that describe best practices for people with ADRD [1, 43]. Diagnosis of MCI due to AD is important to patients and their families, providing opportunities for treatment and future preparations. To have the greatest impact, predictive models should focus on identifying individuals at elevated risk for MCI.

A predictive model for MCI risk based on EHRs could include demographic characteristics (e.g., age, sex, race/ethnicity), biometric data (e.g., blood pressure, body mass index), health-related behavior (e.g., smoking status), laboratory results (e.g., lipids, HbA1c), and the presence of ADRD comorbidities and other data that are accessible in the PCP setting. The model will not include biomarkers, and thus, it will not be a diagnostic tool. However, an easily implemented screening tool for PCPs can greatly improve their ability to identify individuals at elevated risk of MCI. Alerting the PCPs of the possibility of undetected risks could provide an entry point for triage when an individual is flagged for elevated risk. Depending on the maturity of blood-based biomarkers, PCPs could use those results as part of their initial work-up and to decide whether and with what urgency to initiate specialist referrals.

One of the linchpins in the pursuit of the early detection of MCI by PCPs in age-eligible patients is the Medicare Annual Wellness Visit (AWV). Beginning in 2011, the AWV includes the detection of cognitive impairment for Medicare Part B beneficiaries [44]; however, by 2018 uptake of the AWV was still only at 32% [45]. Medicare is primarily available to people aged 65 years or over and this age group is considered the most at risk for MCI and dementia [46]. Being able to identify individuals at risk for MCI before they reach 65 years would enable physicians to treat and track them even earlier, thus potentially limiting the clinical and economic burden of the progression to ADRD.

### Limitations

The results of this study must be considered in the context of several limitations. Firstly, there are those inherent to all claims data: claims data do not allow for proper assessment of potentially relevant clinical variables such as body mass index, smoking status, and the severity, rather than mere presence, of comorbid conditions. Additionally, the generalizability to populations other than the commercially and Medicare supplementally insured, also referred to as Medigap, is unknown. Data from MarketScan is sourced from employers; findings may not be generalizable to the uninsured or underinsured populations. Claims data are collected for reimbursement and not research purposes. This limitation can be addressed by future studies using EHR data, which contains a broader range of predictors and covers an “all-comers” population. An additional benefit to conducting a similar study using EHR data would be the ability to separate individuals with MCI due to AD from the general MCI population, something that was not possible in this study. As mentioned above, because of the underdiagnosis of MCI, this analysis may underestimate the true burden of MCI. The pattern of odds ratio decreasing with increased age may be partially attributed to the expectation that the highest percentage of undiagnosed MCI individuals in the non-MCI cohort is likely to be in the oldest age group. While longitudinal, our observational study design precludes the assessment of causality. Increased diagnoses related to complications as patients near an AD dementia diagnosis have been documented in the literature, which may reflect increased use of health care services as cognitive impairment worsens [47]. Studies that advance our understanding of the diagnostic process, as well as the natural history of the AD continuum, may further elucidate the relationship. In order to address these limitations, studies that compare individuals with diagnosed MCI to individuals with clinically verified normal cognition are needed. In addition, the potential temporal bias could be introduced by using a case-control study design, e.g., patients who saw a doctor more often are more likely to be diagnosed with MCI.

## Conclusions

The ultimate goal of this research is to develop a triage tool to help PCPs identify those with elevated risk of MCI. The 25 ADRD comorbidities were also MCI comorbidities. The comorbidity burden of MCI is likely age-dependent. Based on routinely collected data in the PCP setting, we hope to achieve even better prediction of MCI risk for triage in primary care. This work could enable PCPs to focus on high-risk individuals to initiate further assessment and/or identification of possible MCI and improve the detection of pre-existing MCI in patients who are not voicing concerns about cognitive impairment and initiating an individualized and suitable treatment plan.

### Supplementary Information

**Additional file 1:** **Supplemental Table.** ICD-9 and ICD-10 Codes for Identifying ADRD Comorbidities.

## Data Availability

The data that support the findings of this study are available from the Merative Corporation, but restrictions apply to the availability of these data, which were used under license for the current study, and so are not publicly available. Data are however available from the authors upon reasonable request and with permission of Merative.

## Abbreviations

ADRD: Alzheimer’s disease and related dementia disorders
AUC: Area under the curve
AWV: Annual Wellness Visit
BLLR: Bayesian logistic lasso regression
CCI: Charlson Comorbidity Index
CI: Confidence intervals
ECI: Elixhauser Comorbidity Index
FDA: Food & Drug Administration
EHR: Electronic health records
ICD: International Classification of Disease
IRB: Institutional review board
MCI: Mild cognitive impairment
OR: Odds ratio
PCP: Primary care physicians
Q: Quartile
ROC: Receiver operating characteristic
SD: Standard deviation
TIA: Transient ischemic attack
US: United States
XGBoost: Extreme gradient boosting area
