# Supplementary material for: The age-specific comorbidity burden of mild cognitive impairment: a US claims database study
Source: Alzheimers Res Ther. 2023 Dec 6;15:211. doi: 10.1186/s13195-023-01358-8 (PMC10701954; doi:10.1186/s13195-023-01358-8)
Supplement: Supplementary file 1 — Additional file 1: Supplemental Table. ICD-9 and ICD-10 Codes for Identifying ADRD Comorbidities. [file 13195_2023_1358_MOESM1_ESM.docx]

**Supplemental Table. ICD-9 and ICD-10 Codes for Identifying ADRD Comorbidities**

| **Medical condition** | **ICD-10** | **ICD-9** |
| --- | --- | --- |
| Hypertension, uncomplicated | I10.x | 401.x |
| Hypertension, complicated | I11.x–I13.x, I15.x | 402.x–405.x |
| Stroke / transient ischemic attack | G45.x, G46.x, G97.31, G97.32, I60.xx, I61.x, I63.xx, I66.xx, I67.84x, I97.81x | 428.1,430, 431, 433.x, 434.xx, 435.x, 436, 997.02 |
| Ischemic heart disease | I20.x, I21.0x, I21.xx, I22.x, I23.x, I24.x, I25.xx | 410.xx, 411.x, 412, 413.0, 413.1, 413.9, 414.x |
| Congestive heart failure | I09.9, I11.0, I13.0, I13.2, I25.5, I42.0, I42.5–I42.9, I43.x, I50.x, P29.0 | 398.91, 402.01, 402.11, 402.91, 404.01, 404.03, 404.11, 404.13, 404.91, 404.93, 425.4–425.9, 428.x |
| Myocardial infarction | I21.x, I22.x, I25.2 | 410.x, 412.x |
| Atherosclerosis | I70.0, I70.8, I48.0 | 440 |
| Atrial fibrillation | I48.0, I48.1, 148.11, 148.19, I48.2, 148.20, 148.21, I48.91 | 427.31 |
| Diabetes, uncomplicated | E10.0, E10.1, E10.9, E11.0, E11.1, E11.9, E12.0, E12.1, E12.9, E13.0, E13.1, E13.9, E14.0, E14.1, E14.9 | 250.0–250.3 |
| Diabetes, complicated | E10.2–E10.8, E11.2–E11.8, E12.2–E12.8, E13.2–E13.8 | 250.4–250.9 |
| Hyperlipidemia | E78.0, E78.00, E78.01, E78.1, E78.2, E78.3, E78.4, E78.41, E78.49, E78.5 | 272.0, 272.1, 272.2, 272.3, 272.4 |
| Obesity | E66.x | 278 |
| Metabolic syndrome | E88.xx | 277.7 |
| Weight loss | E40.x–E46.x, R63.4, R64 | 260.x–263.x, 783.2, 799.4 |
| Hearing loss | F80.4, H83.x, H90.x, H91.xx, H92.xx, H93.xx | 389.x |
| Depression | F20.4, F31.3–F31.5, F32.x, F33.x, F34.1, F41.2, F43.2 | 296.2, 296.3, 296.5, 300.4, 309.x, 311 |
| Insomnia | F51.0x, G47.0x | 307.41, 307.42, 327.0x, 780.52 |
| Obstructive sleep apnea | G47.x | 327.xx |
| Disturbances of sensation of smell and taste | R43.2 | 781.1 |
| Bipolar | F30, F31 | 296.0x, 296.1x, 296.4x, 296.5x, 296.6x, 296.7, 296.8x |
| Schizophrenia | F20.x, F25.0, F25.1, F25.8, F25.9 | 295.7x, 295.8x, 295.7x, 297.x |
| Psychosis | F20.x, F22.x–F25.x, F28.x, F29.x, F30.2, F31.2, F31.5 | 293.8, 295.x, 296.04, 296.14, 296.44, 296.54, 297.x, 298.x |
| Alcohol abuse | F10, E52, G62.1, I42.6, K29.2, K70.0, K70.3, K70.9, T51.x, Z50.2, Z71.4, Z72.1 | 265.2, 291.1–291.3, 291.5–291.9, 303.0, 303.9, 305.0, 357.5, 425.5, 535.3, 571.0–571.3, 980.x, V11.3 |
| Drug abuse | F11.x–F16.x, F18.x, F19.x, Z71.5, Z72.2 | 292.x, 304.x, 305.2–305.9, V65.42 |
| Hypothyroidism | E00.x–E03.x, E89.0 | 240.9, 243.x, 244.x, 246.1, 246.8 |
| Chronic kidney disease | B52.0, I12.0, I12.9, I13.0, I13.10, I13.11, I13.2 | 403.9 |
| Chronic pulmonary disease | I27.8, I27.9, J40.x–J47.x, J60.x–J67.x, J68.4, J70.1, J70.3 | 416.8, 416.9, 490.x –505.x, 506.4, 508.1, 508.8 |

Abbreviations: ICD, International Classification of Disease; ADRD, Alzheimer’s Disease and related dementias
